# The Responses of the Ribonuclease A Superfamily to Urinary Tract Infection

**DOI:** 10.3389/fimmu.2019.02786

**Published:** 2019-11-29

**Authors:** Brian Becknell, Christina Ching, John David Spencer

**Affiliations:** ^1^Nephrology and Urology Research Affinity Group, The Abigail Wexner Research Institute at Nationwide Children's Hospital, Columbus, OH, United States; ^2^Center of Clinical and Translational Research, The Abigail Wexner Research Institute at Nationwide Children's Hospital, Columbus, OH, United States; ^3^Division of Nephrology, Nationwide Children's Hospital, Columbus, OH, United States; ^4^Division of Urology, Nationwide Children's Hospital, Columbus, OH, United States

**Keywords:** Ribonuclease A Superfamily, urinary tract infection, innate immunity, pyelonephritis, antimicrobial peptides

## Abstract

The lower urinary tract is routinely exposed to microbes residing in the gastrointestinal tract, yet the urothelium resists invasive infections by gut microorganisms. This infection resistance is attributed to innate defenses in the bladder urothelium, kidney epithelium, and resident or circulating immune cells. In recent years, surmounting evidence suggests that these cell types produce and secrete soluble host defense peptides, including members of the Ribonuclease (RNase) A Superfamily, to combat invasive bacterial challenge. While some of these peptides, including RNase 4 and RNase 7, are abundantly produced by epithelial cells, the expression of others, like RNase 3 and RNase 6, increase at infection sites with immune cell recruitment. The objective of this mini-review is to highlight recent evidence showing the biological importance and responses of RNase A Superfamily members to infection in the kidney and bladder.

## Introduction

Urinary tract infections (UTIs) are one of the most common infections encountered in clinical medicine (1). Nearly half of all women develop one or more UTIs requiring antimicrobial therapy (2, 3). Specific subpopulations have a heightened UTI susceptibility, including pregnant women, people with diabetics, the elderly, people with acquired immunodeficiency diseases, people with structural urologic anomalies, and those who must perform bladder catheterization. Although UTI is not routinely associated with significant acute health morbidities, pregnant women who develop UTI have an increased risk for premature delivery and/or fetal mortality (4). In the elderly, urosepsis is a significant source of mortality (5). Long-term UTI complications include kidney scarring, hypertension, and chronic kidney disease. Thus, UTIs have a significant burden on healthcare resources, with annual costs for UTI management exceeding $2.5 billion United States' dollars (2, 6–8).

Uropathogenic *Escherichia coli* (UPEC), strains of *E. coli* that have adapted to live in extraintestinal niches and cause disease, cause the majority of UTIs (9, 10). UPEC originate from the fecal microbiota, spread across the perineum, ascend the urethra, and invade the bladder. The microbial virulence of UPEC has been linked to many factors that have been previously reviewed (11–13). The most prominent virulence factor are Type I fimbriae, which are adhesion organelles capped by the mannose-binding protein FimH. Type I fimbrae facilitate UPEC attachment to superficial bladder epithelial cells by binding to a matrix of uroplakin proteins (12). After binding, UPEC invade the urothelium and establish a state of commensalism or cause an invasive infection that triggers the activation of innate immune defenses, cellular injury, epithelial proliferation and shedding, cytokine release, and leukocyte recruitment (14). If UPEC ascend from the bladder to the kidney, they concentrate in the collecting duct and attach to the luminal surfaces of intercalated cells. Recent evidence suggests that intercalated cells have a role in UTI defense (15, 16).

To cause a symptomatic infection, UPEC must overcome several innate host defense mechanisms. These include the unidirectional flow of urine and regular bladder emptying that minimize UPEC attachment, alterations in urinary ionic composition that prevent bacterial replication, uroepithelial barrier formation and exfoliation during infection, mucous production, bacterial expulsion, and the secretion of antibacterial peptides and proteins (AMPs) that directly kill invading pathogens or modulate immune defenses (17–19). AMPs that have been identified to prevent UTI include defensins, cathelicidin, lectins, metal binding proteins, and bactericidal peptides of the Ribonuclease (RNase) A Superfamily (20, 21). The following sections of this mini-review highlight published literature investigating the roles of RNase A Superfamily in urinary tract host defense.

## The Ribonuclease A Superfamily

The RNase A Superfamily is a vertebrate-specific gene family that was initially discovered to encode eight human peptides and proteins. These cationic peptides (RNases 1–8) are enzymatically active and can be grouped into four host defense peptide lineages: (1) eosinophil-produced RNases, (2) angiogenins, (3) RNase 6, and (4) RNase 7 and 8 (22–25). Nearly 15 years ago, five additional “non-canonical” ribonucleases were identified (RNase 9–13) that lack a catalytic domain and enzymatic activity (26, 27).

Each canonical RNase A peptide contains a signal peptide and a mature peptide containing 130–159 amino acid residues. Seven of the eight peptides possess eight cysteine residues, forming four disulfide bonds that confer a shared three-dimensional structure across family members. Each peptide also has a conserved catalytic motif (CKXXNTF) (28). Although the canonical peptides are enzymatically active, the catalytic activity may not be necessary for their immunomodulatory or antibacterial functions. While the catalytic motif is conserved, RNase A Superfamily peptides have significant sequence diversity, which may define each peptide's function(s) (21, 28).

Like other host defense peptides, the primary bactericidal mechanism of RNase A peptides is dependent on their ability to disrupt bacterial cell walls. This is driven by the peptide's net charge, amphipathicity, disulphide bonding, and secondary structure (29, 30). The peptide's bactericidal activity is primarily restricted to the amino terminus (31, 32). In addition to their membrane penetrating capability, RNase A peptides can interfere with bacterial attachment, translocate into bacterial cells to inhibit protein and/or DNA synthesis, or initiate signaling pathways important in innate immunity and inflammatory responses (19, 20). As recently reviewed, RNase A Superfamily members can act as chemoattractants, damage-associated molecular patterns (DAMPS or alarmins), immune cell activators, or opsonins. Also, they participate in extracellular RNA clearance (21, 22, 25, 28, 33–35). In the urinary tract, research has primarily focused on their bactericidal activity.

## Epithelial-Produced Ribonucleases

**Figure 1:**
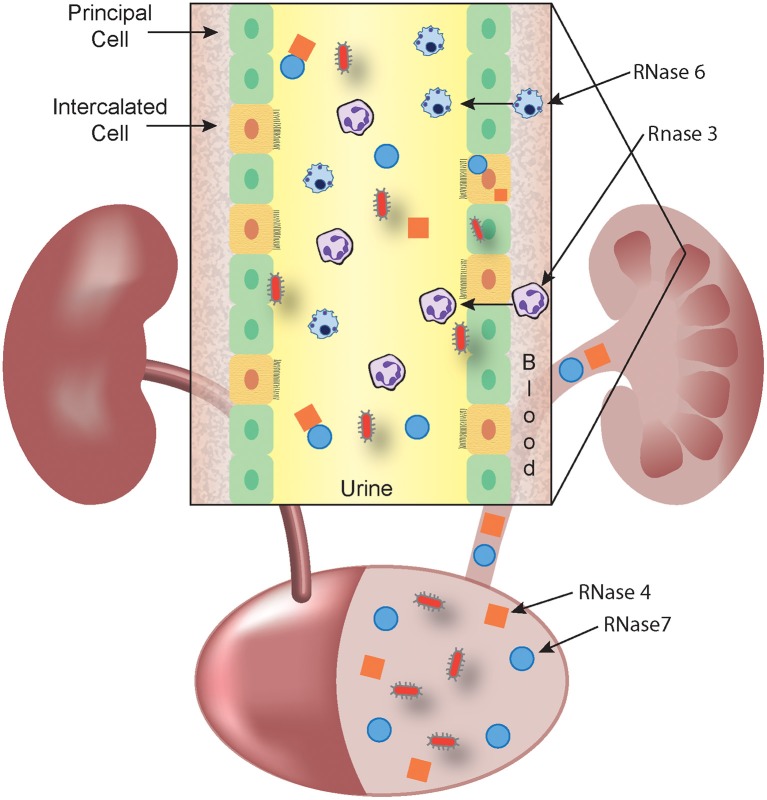
RNase A Superfamily members collaborate to prevent and eradicate UTI. Schematic representation showing that RNase 4 (orange squares) and RNase 7 (blue circles) are produced by the bladder urothelium and the kidney's collecting duct (inset) and released into the urine. In response to microbes (red), circulating leukocytes that harbor RNase 3 (eosinophils and neutrophils) and RNase 6 (monocytes and macrophages) exit the bloodstream and cross the urothelium to accumulate in the urine. The antimicrobial activity of RNase 3 and RNase 6 may be predominantly exerted at the intracellular level, following phagocytosis of microbes. Urinary, parenchymal, and leukocyte-produced RNases kill invading pathogens and facilitate bacterial clearance.

RNase 4 and RNase 7 are produced by epithelial cells in the urinary tract. RNase 7 is produced by the urothelium of the ureter and bladder and secreted into the urinary stream. In the kidney, the collecting duct is the main source of RNase 4 and 7 production (Figure 1) (36, 37).

RNase 4 is one of the least studied members of the Ribonuclease A Superfamily. *RNASE4* mRNA is expressed in multiple human tissues as well as circulating immune cells (38–41). In the urinary tract, the kidney's collecting duct is a source of RNase 4 production where it is regulated by insulin receptor activation and downstream phosphatidylinositol 3-kinase/AKT (PI3K/AKT) signaling (37, 42). RNase 4 is constitutively secreted into the urine and neutralization of urinary RNase 4's antimicrobial activity with RNase 4-specific antibodies facilitates UPEC replication, suggesting it plays a role in UTI defense. Recombinant RNase 4 peptide exhibits antimicrobial activity toward UPEC, including multi-drug resistant UPEC (37). It is possible that monocyte and macrophages are additional sources of RNase 4 in the urinary tract.

RNase 7, which was first identified in human skin, is the best example of an RNase that provides antibacterial defense (43, 44). RNase 7 possesses broad-spectrum antimicrobial activity against Gram-positive and Gram-negative urothopathogens (32, 45). In the urinary tract, RNase 7 is produced by the lower urinary tract and by the kidney's collecting tubules. Within the collecting tubules, RNase 7 is expressed by alpha and beta intercalated cells (36). RNase 7 is secreted into the urine and urinary concentrations increase with UTI to prevent infection (36, 45). Females and adolescent girls with recurrent UTI have suppressed urinary RNase 7 levels compared to controls without UTI (46). When human urine is incubated with RNase 7 neutralizing antibodies and inoculated with UPEC *in vitro*, bacterial growth significantly increases (36, 47). These findings indicate that decreased urinary RNase 7 concentrations are a UTI risk-factor and provide insight into why certain populations are more susceptible to UTI.

RNase 7 expression is restricted to humans and higher vertebrates (26). Genomes of the laboratory mouse or the rat do not contain sequences orthologous to RNase 7, which limited the capacity to assess RNase 7's bactericidal and immunomodulatory functions *in vivo* (26, 27, 30). However, our research group recently generated humanized RNase 7 transgenic mice. RNase 7 expression in these mice did not impact urothelial histology, endogenous innate immune profiles, or the diversity of the gastrointestinal microbiome. When subjected to UTI *in vivo*, these mice were significantly protected from UPEC, suggesting that RNase 7 could be a therapeutic target for protection against UTI (46).

Only limited evidence has evaluated the mechanisms that regulate RNase 7 expression. Published data shows that Toll-Like Receptor (TLR)-mediated pathways, the PI3K/AKT pathway, as well as the mitogen-activated protein kinase (MAPK) pathway regulate RNase 7 expression (48–51). Like RNase 4, our research team has shown that insulin enhances RNase 7 expression via PI3K/AKT activation to shield the urinary tract from UPEC (51). RNase 7's structure, immunomodulatory properties, and bactericidal activity have been recently reviewed (35, 44).

## Leukocyte-Produced Ribonucleases

Eosinophil granule proteins were among the first leukocyte-produced RNases to demonstrate a role in innate immunity (52). Eosinophil Cationic Protein (RNase 3) is a highly basic, 21 kilodalton polypeptide originally isolated from eosinophilic granules (53). In the urinary tract, the expression, immunomodulatory properties, and anti-parasite activity of RNase 3 have been investigated in the setting of schistosomiasis. Urinary RNase 3 concentrations increase during schistosomiasis, and the magnitude of urinary RNase 3 is proportional to the intensity of infection (54, 55). Functionally, purified RNase 3 shows dose-dependent antihelminthic activity toward schistosomula, which represent the larval stage of the *Schistosoma mansoni* parasite (56). Subsequent work demonstrates that RNase 3 exhibits greater antimicrobial activity toward schistosomula than other eosinophilic granule proteins (57). To facilitate schistosomula killing, RNase 3 synergizes with the oxidative burst capacity of plasma membrane fractions derived from neutrophils and eosinophils (58).

While eosinophils are the most studied cellular source of RNase 3, eosinophils generally are not associated with the host response to bacterial UTI. However, studies in the literature argue that neutrophils are also a source of RNase 3, and this cell population is briskly recruited to the urinary space during UTI (59–61). When purified from eosinophils, RNase 3 exhibits antimicrobial activity toward laboratory strains of *Staphyloccus aureus* and *E. coli* (62). To our knowledge, RNase 3's bactericidal activity has not been tested against clinically relevant UPEC strains. Thus, further studies are needed that evaluate RNase 3's expression during bacterial UTI, its cellular source(s) of production, and its contribution to UPEC clearance.

Unlike RNase 3, RNase 6 has been investigated in the context of UTI (24). RNase 6 expression has been localized to human and murine monocytes, macrophages, and neutrophils that are recruited to the urinary tract during UPEC-UTI (Figure 1). Recombinant human and murine RNase 6 peptides exhibit potent, dose-dependent killing of Gram-negative and positive uropathogenic bacteria, comparable to human RNase 7 (24, 63). RNase 6 levels increase in urine from humans and mice with UTI, but its expression was largely retained as an intracellular pool, suggesting that RNase 6 may act intracellularly, following phagocytosis of microbes (24). Further studies are required to determine the contributions of RNase 6 to host immunity during UTI.

## The Non-Canonical Ribonucleases

The less-studied non-canonical RNases, including RNases 9–13, lack the signature catalytic motif of the canonical RNases and do not require enzymatic activity to function (26). These RNases share 15–30% identity with the canonical RNases and contain the signal peptide as well as the three most conserved disulfide bonds. They lack the N-terminal region of mature RNases (21, 28, 64). These RNases are expressed in the epididymis of the male reproductive system and they may regulate sperm maturation and motility (65–67). Recombinant RNase 9 has bactericidal activity against *E. coli* (68). Additional investigation is warranted to define the role of these peptides in UTI defense and the prevention of sexually transmitted infections.

## Discussion and Future Directions

As outlined by the published findings above, significant knowledge gaps remain defining the regulation of antimicrobial activity as well as the roles of antimicrobial RNases—both individually and collectively—in limiting the incidence and spread of UTI. Here, we outline these knowledge gaps and discuss the potential clinical applications of antimicrobial RNases during UTI.

### Mechanisms of Antimicrobial Action

Most published studies of RNase function have focused on the structural elements of each peptide that are required for antimicrobial activity *in vitro* (31, 63, 69, 70). However, it is important that similar structure-function tests be undertaken in the context of eukaryotic cells, which serve as the natural source of RNase A Superfamily members. Recently, we demonstrated that human urothelial cells can be genetically modified to over-express RNase 7, which confers antimicrobial activity toward UPEC (46). This type of experimental approach affords the opportunity to test the relationship between RNase 7 structural elements and antimicrobial function in the context of its cellular source. It is conceivable that this experimental system may reveal key aspects of RNase regulation, including roles for post-translational modifications and protein-protein interactions.

### Regulation of RNase Expression and Function

While emerging data point to the importance of cellular signal transduction pathways such as the insulin-PI3K/AKT pathway as regulators of RNase 4 and RNase 7 in the kidney or bladder, there are significant gaps in our understanding of this process or other processes that are activated during UTI and may impact RNase regulation (37, 51). Also, there are no published data regarding the transcriptional regulation and signal transduction pathways responsible for expression of leukocyte RNases during UTI.

In addition to their regulation at the level of mRNA and protein expression, evidence points to a key role for the RNase Inhibitor (RI) as a regulator of RNase function and cytotoxicity. RI is expressed by all mammalian cells and exhibits high-affinity binding with multiple members of the RNase A Superfamily (47, 71). RI complexes to RNase A proteins in the cytosolic compartment, attenuating or inhibiting their biological functions and protecting host cells from their cytotoxic RNase activity. Raines and colleagues have elegantly shown that RNase A peptides complexed to RI have limited cellular toxicity. In contrast, RI-elusive RNases degrade cellular RNA and trigger apoptosis (72, 73). Currently, there is only limited data evaluating the role of RI in the kidney or urinary tract. While no studies have evaluated the impact of RI on urothelial proliferation or cellular protection during UTI, we have shown that RI complexes to recombinant RNase 7 and abrogates it's antimicrobial activity against uropathogens (47). Similarly, RI complexes to RNase 7 in kidney tissue as well as in the urine sediment *in vivo*. In the context of acute or chronic UTI, the significance of this interaction remains unknown.

Specifically, are there events triggered by infection that modify the interaction between RNase 7 and RI? The interaction between RNase 7 and RI is dependent on the presence of intact disulfide bridges in RI, as disruption of these bridges triggers rapid dissociation of the RNase 7:RI complex (47). This has led us to speculate that the RNase 7's antimicrobial activity may be redox dependent (47). There is precedent for this among other AMPs, such as human β-defensin 1 and paneth cell α-defensin 6 (74, 75). Alternatively, we have found that neutrophil proteases can degrade RI, and this may be a mechanism for recruited inflammatory cells to augment local RNase activity during UTI (47). The precise roles of RNase:RI interactions and their regulation during UTI remain a significant knowledge gap. As research progresses, the benefits and risks of RI-interactions and evasion need to be carefully evaluated.

### Roles of RNases During UTI *in vivo*

While the antimicrobial activity of RNases can be measured *in vitro*, the ultimate biological test of relevance is to over-express and delete antimicrobial RNases and demonstrate a consequence on host susceptibility to experimental UTI in laboratory animals. These experiments are challenging, since there are instances in which genes encoding human antimicrobial RNases have no orthologous mouse gene (*RNASE7*) or up to 15 paralogous mouse genes (*RNASE3*) (76). Moreover, there are instances in which the same cell type expresses multiple antimicrobial RNases, such as monocytes in the case of RNase 4 and RNase 6, or epithelial cells in the case of RNase 4 and RNase 7 (36, 37, 41, 77). These circumstances can lead to gene redundancy and lack of an overt phenotype when a single gene is deleted. Thus, we have taken the opposite approach, namely, to generate humanized *RNASE7* transgenic mice. This gain of function experiment has allowed us to establish the consequences of *RNASE7* expression on host immune functions and susceptibility to experimental UTI *in vivo* (46). Further studies are required to determine the function of RNases—both individually and collectively—in the setting of UTI, as well as to determine their mechanisms of action.

### Impact of Uropathogens on RNase Expression and Activity

Up to this point, we have addressed the impact of RNases on microbes as unidirectional. However, it is likely that uropathogens influence the levels and activity of RNase peptides. For example, UPEC strains expressing the toxin Hemolysin A (HlyA) attenuate intracellular PI3K/AKT signaling, leading to decreased RNase 7 expression *in vitro* (51). Similarly, UPEC produced proteases like OmpT have the ability to degrade RNase 7 (78). Thus, additional studies are required to identify the ways in which microbes might regulate the expression and activity of antimicrobial RNases in the urinary tract.

### Applications of RNases as Therapeutics

Interest has centered on use of AMPs as an antibiotic-independent mechanism of limiting, treating, and even preventing UTIs. This interest has been fueled by a growing concern regarding antibiotic overuse and its impact on rising bacterial antibiotic resistance rates (20, 79). AMPs may offer an alternate therapeutic strategy, with even some benefit over antibiotics given their efficacy at low concentrations, limited bacterial resistance patterns, and potential synergistic mechanisms of action with conventional antibiotics (19, 80–82).

However, multiple hurdles must be overcome to realize the direct therapeutic potential of RNase as AMPs in patients with UTI. This includes achieving the synthesis of large quantities of purified RNase peptides or peptide fragments, taking steps to assure stability and delivery, and ensuring that RNases do not exhibit significant toxicity toward host cells. Despite these formidable challenges, we envision future direct applications of antimicrobial RNases to people with highest UTI risk. In addition to such direct applications, we also envision circumstances in which endogenous pathways are pharmacologically triggered to induce RNase expression as an alternative strategy to prevent and even treat UTI. In sum, while many knowledge gaps remain, the urinary tract is an excellent venue to realize the biological significance and therapeutic applications of antimicrobial RNases and define their roles as novel therapies.

## Author Contributions

BB, CC, and JS performed the literature review and wrote the manuscript together.

### Conflict of Interest

The authors declare that the research was conducted in the absence of any commercial or financial relationships that could be construed as A potential conflict of interest.
